# The efficacy of a photolyase-based device on the cancerization field: a clinical and thermographic study

**DOI:** 10.1186/s13046-015-0203-0

**Published:** 2015-08-19

**Authors:** Luigi Laino, Fulvia Elia, Flora Desiderio, Alessandra Scarabello, Isabella Sperduti, Carlo Cota, Aldo DiCarlo

**Affiliations:** Thermography, San Gallicano Dermatologic Institute for Research and Care, Via Elio Chianesi 53, 00144 Rome, Italy; Radiology, San Gallicano Dermatologic Institute for Research and Care, Via Elio Chianesi 53, 00144 Rome, Italy; Clinical Dermatology, San Gallicano Dermatologic Institute for Research and Care, Via Elio Chianesi 53, 00144 Rome, Italy; Biostatistical Unit, IFO-Regina Elena National Cancer Institute for Research and Care, Via Elio Chianesi 53, 00144 Rome, Italy; Dermopathology, San Gallicano Dermatologic Institute for Research and Care, Via Elio Chianesi 53, 00144 Rome, Italy; Scientific Director, San Gallicano Dermatologic Institute for Research and Care, Via Elio Chianesi 53, 00144 Rome, Italy

**Keywords:** Cancerization field, Active telethermography, Hyperthermic halo, Skin cancer

## Abstract

**Background:**

At skin level, a cancerization field (CF) indicates some chronically photoexposed areas in which, besides a primary tumor, histological or biomolecular modifications without clinical signs are present. Active telethermography (ATT) allows us to observe the imaging of a hyperthermic halo (HH) surrounding the tumor . The Authors hypothesize HH as a possible expression of CF.

**Objectives:**

The aim of this study were to verify whether HHs have the same histological or immunohistochemical characteristics as the CF and, secondly, to evaluate the efficacy of a device containing the enzyme photolyase in modifying thermographic parameters in these area.

**Methods:**

The study included 30 patients affected by actinic keratosis, evaluated clinically and by ATT at time 0 and after 3, 6 and 9 months.

**Results:**

The ATT showed the presence of HHs in all the patients and, after the treatment, a significant modification of both the extension of these areas and the thermal parameters. In 5 patients for whom, while operated, two other biopsies were performed, respectively on the HH and on a perilesional non-hyperthermic area, in the HH, we detected a p53 and Ki 67 over-expression.

**Conclusions:**

Our results indicate that ATT could represent a useful paraclinic method in identifying CFs in subjects at risk.

## Introduction

Actinic keratosis (AK) is one of the most frequent skin tumors, affecting in particular the Caucasian population [1–4]. Such lesions are mainly located in sun-exposed areas, i.e. head, face, and arms. Elderly people, in particular farmers and sailors, as well as individuals who have undergone immunosuppressive treatments or are chronically exposed to arsenic and tar, are at increased risk of AK [5, 6]. Some important co-factors are, the skin phototype (type I and II according to the Fitzpatrick classification), baldness, genetic predisposition, sun exposure or burns in childhood and UV-A treatments [7–10]. Clinically, AK is characterized by erithematous or brown lesions with a diameter of less than 1 cm, showing a hyperkeratotic surface, sometimes erosive, and well-defined edges. Histologically, AK is an *in situ* squamous cell carcinoma (SCC) and its progression to SCC ranges from 0.25 to 16 % of cases, as reported in the literature [11–13]. AK lesions are often not unique, and patients may experience new lesions in the same area, which can appear concomitant or later, in the course of the years. Moreover, in this region, the development of other malignant skin tumors such as SCC or basocellular carcinoma (BCC), after the primary AK, is also possible. This particular behavior corresponds to the concept of “cancerization field” (CF), as proposed by Slaughter in 1953 [14]. As a general characteristic, CF indicates the risk of having a second tumor in the same area after the removal of the first one. This preneoplastic condition, described initially in the oral mucosa, has been successively reported in internal organs and in the skin. At the cutaneous level, CF is addressed to the chronically photoexposed area of the head, particularly in the fair-skinned individuals that, after a first diagnosis of AK or other non-melanoma skin cancer (NMSC), can in the future present one or more malignant lesions in the same site. Indeed, according to Slaughter [14], other Authors found some peculiar histological and biomolecular modifications in these critical areas, even in the absence of clinical signs. In fact, at the histological level, there have been reports of loss of cell polarity, disorganization, and hyper- and para-keratosis associated with dermal modifications (elastosis, vascular ectasia, reduction of collagen fibers) [15]. Immunohistochemically, some mutations of important genes e.g. p53, Bcl 212, Ki 67, p21, CPI-17, PCNA, MMP-1, and also alterations of pro-collagen 1 and tenascin-C have been demonstrated [16, 17]. In addition, in experimental models, some Authors have described, the production of a high number of cyclobutane pyrimidine dimers (CPD), the major DNA photoproducts of UV radiations, in the CF [18].

Given the foregoing, the study of CF is very important for the monitoring of individuals already treated for AK, with the aim of preventing a second tumor. To this purpose, many non-invasive methods have been employed in order to identify these areas at risk, such as dermoscopy and confocal microscopy [19, 20]. Also, mathematical models have been proposed [21]. Telethermography (TT) is a non-invasive method that collects the infrared radiation emitted from the body and creates an electronic image [22, 23]. Since its first description by Brasfield [24], many Authors have observed that malignant tumors of the skin, such as melanoma and SCC, show a “hot” pattern, while benign lesions (e.g. seborrheic warts), and BCC appear as “cold” [24, 25]. In those area, thermal values, particularly in large nodular forms, could reach even 3-4 °C. However, if the tumoral lesions are small (e.g. diameter <1 cm), they could be thermographically undetectable or “missed” (as Brasfield first noted) [24], particularly when the lesions are sited in physiological “hot” regions (e.g. groin, axilla) (false negatives). The principle of dynamic or active telethermography (ATT) is that, by cooling or heating by air or by gel packs the skin for a defined period of time, is possible to better observe the specific pattern of the tumor. This pattern appears rapidly (in seconds) in the course of the thermal recovering of the area after the removal of the stimulation, in contrast with the thermal recovery of the perilesional healthy skin (in minutes) [25, 26]. The thermal recovery time (TRT) is the interval between t^0^ and the time that single thermal points come back to the steady state (expressed in seconds, or minute). The first advantage of the method is the possibility to evaluate very small malignant lesions that, having a reduced critical mass, are difficult to observe using direct thermography. Furthermore, thermostimulation does not require climatizing of the operating room, the thermal recovery being independent of the normal working conditions of the room. In melanoma studies, we found TT useful for the diagnosis of in-transit metastases from melanoma [27, 28]. Some methods use a periodic thermal modulation, e.g. lock-in thermal imaging (LIT) [29].

Introducing the “thermostimulation” method in 1995, our intent was to perform a thermal stress under controlled conditions, and following the transient temperature measurement [30]. By selecting the infrared imaging at a specific time after thermostimulation, it is possible to “reject” the effect of local steady state and to select the peculiar thermographic pattern of the lesions. For this procedure, a special equipment has been adopted, namely the thermostimulator (Reg. Italy. Patent 1323870/IT/2001) [29] (see Methods). In a recent paper, we demonstrate by using ATT that AK has not only a “hot” pattern and a rapid TRT but it is surrounded by a hyperthermic halo (HH), whose thermal intensity (Δt) appears lower than that of the tumor. This HH expands generally for 2–4 cm^2^ around the clinical lesion [30].

On these premises, our hypothesis was that HH could correspond to the CF. To demonstrate this, the aim of this study was to verify whether, in the HH area were present, at histological and immunohistochemical level, the same changes present in the CF, as reported in literature [30–32]. Furthermore, we proposed to verify by this method the efficacy of the compound Eryfotona^R^ in reducing the degree and the extension of the HH. Eryfotona is a film-forming medical device in fluid formulation containing a DNA-repair enzyme photolyase and a high-protection UV filter (Repairsomes™) that is indicated in the treatment of CF in patients affected by AK. Previous studies have shown that Eryfotona is effective in treating and preventing the abnormal histological and biomolecular cellular pattern of CF [31–35].

## Patients and methods

### Patients and Eryfotona treatment

A group of 30 patients affected by AK, respectively 27 males and 3 females, referred to our Institute in the period January-April 2013. The age ranged from 55 to 75 years (mean = 64.3 years). All the patients had phototype I-II, and a personal history of prolonged exposure to sun rays, for professional or recreational reasons-respectively 14 farmers, 4 sailors, 4 lifeguards, while the other 8 lived near the sea. Most of them (27/30 patients) had baldness at an advanced stage, while all female patients had androgenic alopecia with severe baldness in the temporo-frontal region. The AK lesions were mainly sited in the temporo-parietal, frontal and occipital regions.

Treatment with Eryfotona started 15 days after surgical removal of the lesions. Eryfotona was applied daily (at 8 and 12 h) by the patient in the areas concerned, at a standard dose for 9 months. Written informed consent was obtained from all patients, and the study was approved by the institutional research board and conducted according to the Declaration of Helsinki Principles. Three drop-outs for poor compliance (Patients n 7, 8, 10) were reported.

### Active telethermography (ATT)

For this procedure we employed a FLIR3000 Thermocam™ together with the thermostimulation method. We used a special equipment, namely the thermostimulator (Reg. Italy. Patent 1323870/IT/2001) [29] consisting of a small insulated tank containing 5 l of a mixture of alcohol and water at 50 % and connected through a two-way tube to the probe, a rubber balloon with a capacity of 250 ml. The mixture can be led to a preset temperature, by means of a heating-cooling system put inside the walls of the tank (range possibility: from 0 °C to 40 °C). The temperature of the liquid in the container and the time of application are displayed on monitors. After the mixture reaches the set temperature (in our study: +5 °C), the liquid is driven at high speed by means of the pump to fill the balloon. Then, the balloon is rested on the skin for 20 s. The moment when stress is removed (= t^0^), the whole stimulated skin area has homogenously a thermal value of +5 °C. The area of the hyperthermic halo (HH) is automatically calculated by a simple function of the PC connected with the thermocamera. To ensure that the same skin surface is observed at each examination, we used a specific function of the PC connected to the thermocamera, and in case of several lesions, a cutaneous marker was used. Thermal recovery time (TRT) is the interval between t^0^ and the time that single thermal points come back to the steady state (expressed in seconds, or minute). Clinical and thermographic evaluations were planned respectively at time 0, and 3, 6 and 9 months of treatment. TRT was considered as rapid (R) for values <10 s, slow (S) for values >10 s and < 2 min, and normal (N) for values of healthy perilesional skin (>2 min).

### Immunohistochemistry

In 5 of the subjects (n = 30), two punch biopsies were also performed during the surgical removal of AK, respectively, one in the area of HH and the other in a perilesional non-hyperthermic area. All specimens were processed using the standard histopathologic method of paraffin embedding sectioning and hematoxylin-eosin (HE) staining. In these 5 patients, in addition to histological evaluation, an immunohistochemical test for p53 and Ki67 was also conducted.

### Statistical analyses

Descriptive statistics were used to represent study results as percentages and medians (range). A Friedman test for non-parametric data was used to test changes in each variable over time, while a McNemar test was used for paired data. The SPSS (21.0) statistical program was used for analysis.

## Results

**Fig. 1:**
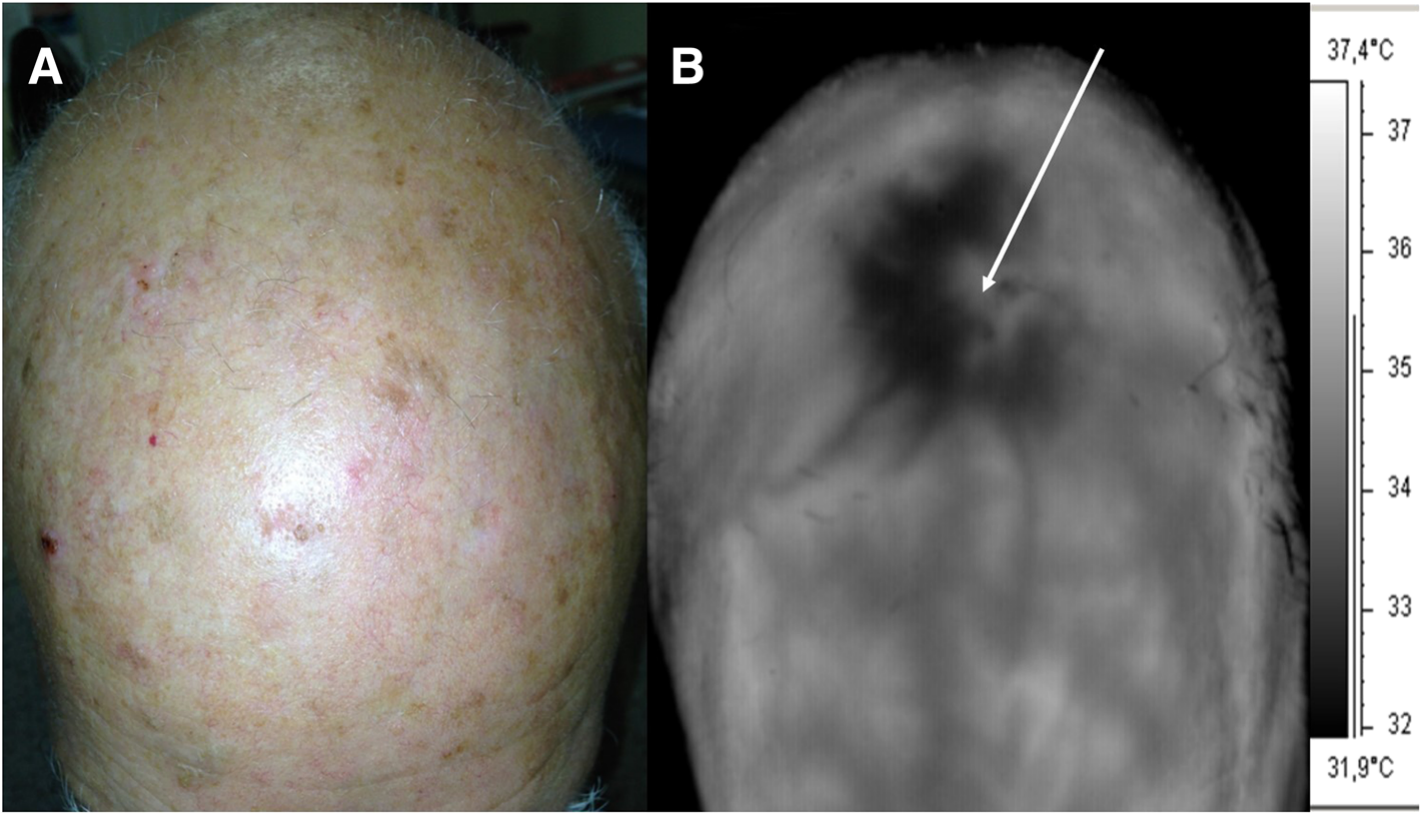
**a** AK of the scalp. **b** TTG of the scalp. Note the hyperthermic “halo”, while squamous crusts make the AK lesion itself apparently “cold”

**Fig. 2:**
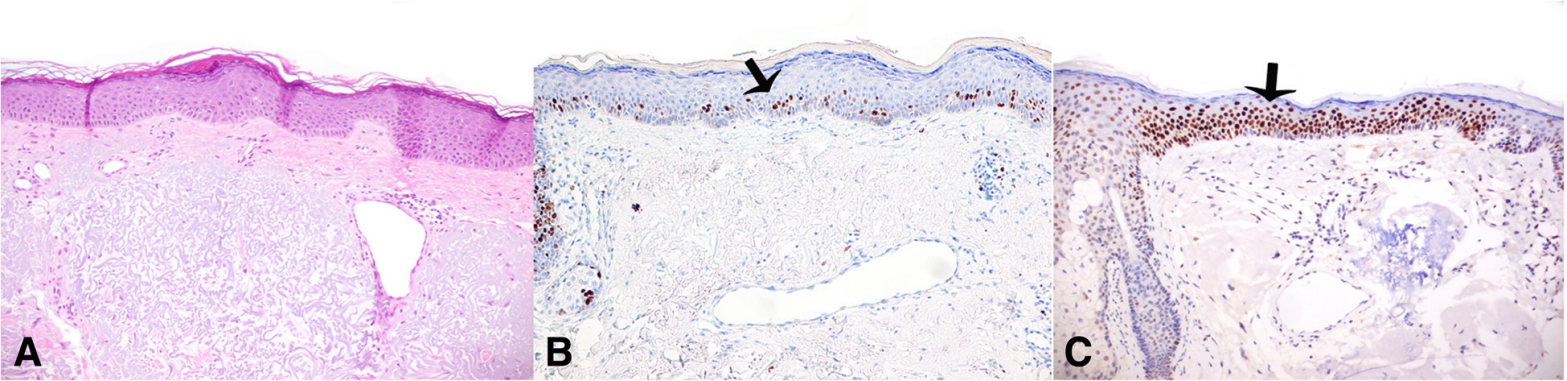
**a** Histologic examination of skin biopsy from cancerization field. The epidermis appears normal without evident atypia of the keratinocytes. In the dermis, marked actinic elastosis is present. **b** Proliferating cell in suprabasal layers, as marked with Ki67. **c** p53 expression of the epidermis in corresponding area of cancerization field (arrow)

The thermographic presence of HH was observed in all the patients before the surgical removal of AK. These HHs extended from few mm^2^ to some cm^2^ (mean 3.46 cm^2^), and their TRTs, very rapid at baseline, showed generally progressive increase at each control (Fig. 1 and Table 1). In the 27 patients that completed the study, after treatment with Eryfotona we observed that the area of the HHs declined from a mean of 3.46 cm^2^ at baseline to a mean of 0.64 cm^2^ at 9 months, while the values of TRT progressively increased toward the perilesional values of the healthy skin (>2 min) (Table 1). In 5 cases, the HH disappeared completely. In parallel, at each periodic control, the TRT values showed a progressive tendency to the same values as the perilesional healthy skin. In those 5 patients in which a double biopsy was performed, in the HH site, we observed, histologically, a more or less marked actinic elastosis and p53 and Ki 67 overexpression in the epidermis (Fig. 2). These data were less evident or absent in the non-hyperthermic perilesional site (data not shown).

**Table 1:** Evolution of the hyperthermic halo (HH) in 27 patients. The diagram shows, respectively, its extension (in cm^2^) and RTR

		Baseline		I Check (3 Months)		II Check (6 Months)		III Check (9 Months)	
Patient	Lesion site	HH Area	TRT ^(a)^	HH Area	TRT	HH Area	TRT	HH Area	TRT
		(cm^2^)		(cm^2^)		(cm^2^)		(cm^2^)	
1	Frontotemporal	5	R	4	R	2	R	1	S
2	Frontoparietal	4	R	3	R	1	S	0,5	S
3	Frontoparietal	4	R	3	R	1	R	0,5	S
4	Temporal	3	R	2	R	1	S	1	S
5	Frontotemporal	3	R	2	R	1	R	0	N
6	Occipital	5	R	4	R	1	R	0	N
7	Occipital	4	R	3	R	Drop-out			
8	Occipital	3	R	2	R	2	R	Drop-out	
9	Temporal	5	R	3	R	1	R	1	S
10	Frontoparietal	4	R	3	R	Drop-out			
11	Occipital	3	R	2	S	1	S	0	N
12	Frontal	2	R	2	R	1	S	1	S
13	Parietal	5	R	4	R	3	R	1	S
14	Occipital	3	R	2	R	1	R	0,5	S
15	Occipital	2	R	1	R	0,5	N	0	N
16	Frontotemporal	3	R	2	R	1	N	1	R
17	Frontoparietal	4	R	3	R	2	R	1	R
18	Parieto-temporal	2	R	1	R	1	R	0,5	S
19	Temporal	4	R	3	R	2	N	0,5	S
20	Frontotemporal	4	R	3	R	2	R	0,5	S
21	Temporo-occipital	2	R	1	R	1	R	0,5	S
S22	Frontoparietal	3	R	2	S	1	S	1	S
23	Occipital	4	R	3	S	2	S	1	R
24	Temporal	5	R	4	R	3	R	0,8	SN
25	Frontoparietal	3	R	2	R	1	S	0,5	SN
26	Occipital	2	R	1	S	0,5	S	0,5	S
27	Frontal	2	R	1	R	1	S	0,6	S
28	Parietal	3	R	2	R	1	R	0,5	S
29	Occipital	4	R	3	R	2	R	1	S
30	Frontal	4	R	3	R	2	R	0	N

^(a)^TRT = Thermal recovery times. Rapid (<10 s): Slow (>10 s < 2 min); Normal (>2′)

## Discussion

The term “cancerization field” (CF) was initially introduced by Slaughter for describing the presence in the oral cavity of some histological alterations besides or near the primary tumor, in the absence of clinical evidence. For this reason, he expressed the concept of CF as a “benign epithelium preconditioned to change toward cancer” [14]. The possibility of a gradual progression from subclinic condition to a clinically detectable SCC was also confirmed by other Authors [15–18]. At skin level, the most significant example of CF is considered the sun-exposed areas of individual at risk, in which also AKs and other NMSC are frequently observed. Among non-invasive methods proposed to identify these areas, the ATT method uniquely allow us to observe in imaging the presence of the HH that could be the visible expression of CF. Thermographically, HH is defined as a hyperthermic halo surrounding the AK lesion. Its identification is possible by means of ATT that shows its quick thermal recovery and its particular morphology (asymmetric hyperthermic halo). Introducing the “thermostimulation” method in 1995, our intent was to perform a thermal stress under controlled conditions, and following the transient temperature measurement [29]. In our previous studies, based always on the same parameters (+5 °C × 20″), we report a value of TRT >2 min in the case of healthy skin, in agreement with others [25, 26]. On an average evaluation, the thermal difference among tumor, HH and perilesional healthy skin, are often <0.01 °C, leading to “miss” these hot spot being low the current accuracy/sensitivity of the ATT devices for this purpose. The thermostimulation method let us to easily overpass the problem of the direct ATT by switching the thermographic static values (in °C) with the times of recovery expressed in min/s. TRTs in case of malignant tumor are in general of the order of second, while those of the healthy are in the order of minutes, according to our parameters of thermal stress (+5 °C × 20”). In our observations, HH has a less rapid thermal recovery respect to the tumor as it is reported in the Table 1. In the case of malignant tumors of the skin, such as SCC and malignant melanoma, we observe, few seconds after t^0^, the thermal image of the tumor as a hot spot, while residual field is still cold [25, 26]. However, in cases of basocellular carcinoma, we have noted a long persistence of hypothermia after the thermostimulation (an order of minutes) as happens also for other pigmented benign lesions (e.g. seborrheic warts) [29]. The molecular mechanisms of HH are not clear. Possible explanation has be suggested to be the high esothermic metabolism of the malignant tumors in contrast to other lesions such as basalioma [26]. In agreement with this hypothesis, we found, in the 5 cases in which we performed the perilesional biopsies, overexpression of Ki 67 and p53, the latter indicative of gene mutation, particularly in the correspondence of HH that is suggestive of tumor transformation. Recent studies in molecular genetics hypothesize a carcinogenetic model based on the clonal expansion of genetically modified cells following the chronic action of UV radiations. In particular, DNA-modifying UV-A and, to greater degree, UV-B and RNA structure foster the formation of CPD with subsequent mutations of the telomerase or oncosuppressor p53 gene [19]. However, to confirm our hypothesis, it is necessary to have more data, in particular, a larger number of patients and samples.

The reason for the enrolment of a small number of patients to evaluate the histological and immunohistochemical studies of HH (5 subjects) was due to the fact that the principal end-point of this paper was to confirm by means of ATT the presence of HH in all the patients affected by AK. Then, we performed the immunohistochemical tests only in those patients that consented to more biopsies. Eryfotona^R^ proved effective in reducing the extension of HHs and their thermal values in our 27 subjects after a treatment of 9 months. The mechanism of action of Eryfotona is dual, a preventive one of the UV-block by means of a strong sunscreen and a molecular enzymatic action on DNA (photolyase). Furthermore, topical application of liposome formulations with CPD photolyases onto human skin provides protection against UVB-induced damage [36-37]. Finally, patients declared, from the first control, a sharp reduction, in most cases a complete resolution, of the itchiness and/or pain referred to the lesional and perilesional area.

In conclusion, among the non-invasive methods from time to time proposed in the study of CF, ATT could represent a useful paraclinic method in identifying CF in subjects at risk. Thermography has a long story in clinical applications, but thermal imaging in dermatology is not much employed and the papers on this issue are very few among dermatologists, the specialists that could benefit most in this field. This method is easy to use, gives an immediate response, and is repeatable and reproducible. For this reason, ATT could be very useful for epidemiological controls in some groups at risk, in the follow-up of operated patients, and in the assessment of the efficacy of proposed treatments.

## Abbreviations

AK: Actinic keratosis
ATT: Active telethermography
BCC: Basocellular carcinoma
CF: Cancerization field
CPD: Cyclobutane pyrimidine dimmers
HE: Hematoxylin-eosin
HH: Hyperthermic halo
LIT: Lock-in thermal imaging
NMSC: Non-melanoma skin cancer
SCC: Squamous cell carcinoma
TRT: Thermal recovery time
